# Rituximab in Refractory Myositis and Acute Neuropathy Secondary to Checkpoint Inhibitor Therapy

**DOI:** 10.7759/cureus.25129

**Published:** 2022-05-19

**Authors:** Varun Jain, William Remley, Cyra Bunag, Aisha Elfasi, Miguel Chuquilin

**Affiliations:** 1 Department of Neurology, University of Florida College of Medicine, Gainesville, USA; 2 Department of Neurology, Lake Erie College of Osteopathic Medicine, Jacksonville, USA; 3 Department of Neurology, University of Florida, Gainesville, USA

**Keywords:** neuropathy from checkpoint inhibitor therapy, myositis from checkpoint inhibitor therapy, refractory neurological complication from check point therapy, rituximab in myasthenia from ipilimumab, check point therapy complication

## Abstract

Checkpoint inhibitor immunotherapies have been one of the latest advances through the last decade in the treatment of various cancers. As their use is increasing so is the knowledge of their complications which can affect various organ systems including the central and peripheral nervous systems. Management of these complications requires stopping the offending agent and in some cases treating with immunosuppressive agents like intravenous steroids. Physicians can face challenging situations if patients are unresponsive to steroids, intravenous immunoglobulins (IVIG), and plasmapheresis (PLEX). There are no formal guidelines to help in the management of such patients.

We present an 85-year-old male with a past medical history of renal cell carcinoma status post nephrectomy who was admitted with diplopia, eyelid ptosis, dysphagia, dysphonia, dyspnea, and generalized weakness. He was started on nivolumab and ipilimumab 10 days prior to presentation. Laboratory studies showed an elevated erythrocyte sedimentation rate, C-reactive protein, and creatine phosphokinase, and an unrevealing lumbar puncture. Acetylcholine receptor (AChR) and muscle-specific kinase (MuSK) antibodies were negative. Electromyography and nerve conduction studies showed axonal and demyelinating sensorimotor neuropathy with no significant decrement on 3 Hz repetitive stimulation. Thyroid function tests were concerning for thyroiditis and anti-thyroid peroxidase antibodies were positive. Together, these findings led to the diagnosis of refractory myositis and acute neuropathy along with autoimmune thyroiditis from nivolumab and ipilimumab immunotherapy. His symptoms were unresponsive to a 5-day course of steroids, intravenous immunoglobulins, and plasmapheresis. He was then started on rituximab with significant improvement in ptosis, dysphagia, dysphonia, and proximal weakness. Immune checkpoint inhibitors (ICI) are associated with an increased risk for the development of various autoimmune conditions. Treatment involves discontinuation of the offending drug and initiation of immunosuppressive therapy.

This case is interesting as it demonstrates the importance of the awareness of the neurological complications of the checkpoint inhibitor therapies and the beneficial role of rituximab in patients who are unresponsive to initial immunosuppressive therapies including steroids, IVIG, and PLEX.

## Introduction

Checkpoint inhibitor immunotherapy has a wide footprint of use in the world of oncology. As its list of indications gets longer, so do the adverse effects witnessed by its use [1]. So far steroids and intravenous immunoglobulins (IVIG) have been helpful in treating most of the inflammatory adverse effects [2]. There is not enough data to guide the management of the adverse effects that are refractory to steroids and IVIG. Our patient who presented with new-onset neurological deficits after ipilimumab and nivolumab treatment was diagnosed with acute myositis and neuropathy but was unresponsive to steroids and IVIG therapy. We used rituximab and achieved remission of most of his symptoms. More studies are needed to validate the use of rituximab as a treatment for neurological adverse effects from checkpoint inhibitor therapy. 

## Case presentation

An 85-year-old male presented to the hospital with diplopia, bilateral eyelid ptosis, dysphagia, dysphonia, and shortness of breath for two days. He had a history of renal cell carcinoma status post left nephrectomy and coronary artery disease with stent placement. He had started ipilimumab and nivolumab 10 days prior. He denied arthralgia, rash, fever, chest pain, or recent weight loss. 

On physical examination, he had binocular horizontal diplopia, bilateral eyelid ptosis, bilateral horizontal and vertical ophthalmoplegia, neck flexion weakness, facial diplegia, weak tongue protrusion, asymmetric proximal upper limb muscle weakness, and right foot drop. His vital capacity and negative inspiratory force were normal. The rest of his physical exam, including mental status, language, sensation, and reflexes, was normal.

Given the above symptoms, differential diagnoses considered were neuromuscular junction diseases (myasthenia gravis, Lambert-Eaton myasthenic syndrome, botulism), inflammatory myopathy/myositis, and peripheral neuropathy (a pharyngeal-cervical-brachial variant of Guillain-Barre syndrome). Inflammatory disease (neurosarcoidosis) and neoplastic process ( central nervous system (CNS) lymphoma, leptomeningeal disease) were also considered.

Complete blood count and basic metabolic panel were unremarkable. As shown in Table 1, erythrocyte sedimentation rate (ESR) and high sensitivity C-reactive protein (CRP) were elevated indicating an inflammatory reaction. Increased creatine kinase (CK) and mild transaminitis were probably due to underlying myositis. The thyroid panel was significant for elevated thyroxine (free T4) levels, decreased thyroid-stimulating hormone (TSH) levels, and positive thyroid peroxidase antibodies (TPO Ab) reflecting autoimmune thyroiditis. Cell count, chemistries, and cultures of spinal fluid were unremarkable. Acetylcholine receptor antibodies profile and muscle-specific kinase antibodies were not significant. A paraneoplastic panel of anti-Yo, anti-Ri, and anti-Hu antibodies was also unrevealing. Electromyography/nerve conduction study (EMG/NCS) showed axonal and demyelinating sensorimotor neuropathy with no significant decrement on 3 Hz repetitive stimulation. Myopathic changes were absent.

**Table 1:** Laboratory Results

Labs	Results	Units	References
White Blood Cells	5.06	per μl	4-10.8
Hemoglobin	11.7	g/dL	13.5-16
Hematocrit	34.3	%	41-49
Platelets	229	per μl	130-240
Aspartate Aminotransferase	216	U/L	0-45
Alanine Transaminase	109	U/L	0-45
Creatine Phosphokinase	2408	U/L	5-180
Erythrocyte Sedimentation Rate	57	mm/hr	0-10
C-Reactive Protein	11	mg/L	0-5
Thyroid Stimulating Hormone	0.02	mIU/L	0.4-5
Free Thyroxine (T4)	3.1	ng/dL	0.6-1.2
Free Triiodothyronine (T3)	0.5	ng/dL	0.8-2.0
Thyroid Peroxidase Antibody (TPO Ab)	43.4	IU/mL	<9.0
Acetylcholine Receptor (AchR) Binding Antibody	0.2	nmol/L	0.0-0.4
Acetylcholine Receptor (AchR) Blocking Antibody	15	% inhibition	0-26
Acetylcholine Receptor (AchR) Modulating Antibody	15	% inhibition	<32%
Muscle Specific Kinase (MuSK) Antibody	Negative		Negative
Hepatitis C Antibody	Negative		Negative
Hepatitis Bs Antigen	Negative		Negative
Hepatitis Bs Antibody	Negative		Negative
Hepatitis B Core Antibody	Negative		Negative
Lyme Enzyme Immunoassay	Negative		Negative
Anti Yo Ab	Negative		Negative
Anti Hu Ab	Negative		Negative
Anti Ri Ab	Negative		Negative
SPINAL FLUID STUDIES			
White Blood Cells	8	cells/cmm	0
Red Blood Cells	7	cells/cmm	0
Proteins	59	mg/dL	15-45
Glucose	88	mg/dL	40-70

The absence of decrement with 3 Hz repetitive stimulation and negative acetylcholine receptor and muscle-specific kinase (MuSK) antibody panel ruled out myasthenia gravis. Given no prodrome of fever, chills, unremarkable spinal fluid cell count and chemistries, and negative spinal fluid polymerase chain reaction studies made the likelihood of CNS infection and primary CNS vasculitis less likely. Normal brain MRI with and without contrast ruled out CNS malignancy, metastatic disease, and inflammatory disease such as neurosarcoidosis. Flow cytometry and cytology of spinal fluid were also negative. Chest X-ray did not show hilar lymphadenopathy. Elevated ESR, CRP, and creatine phosphokinase​​​​​​​ (CPK)raised suspicion for myositis syndrome but muscle biopsy could not be obtained to confirm it.

The patient was diagnosed with acute myositis, acute axonal and demyelinating sensorimotor neuropathy, and autoimmune thyroiditis from checkpoint inhibitor therapy. He did not respond to methylprednisolone 1 gram/day intravenously for five days, so the treatment was escalated and he received intravenous immunoglobulins (IVIG, 2 g/kg) over five days. Given the lack of any response to steroids and IVIG and the persistence of his neurological symptoms, he underwent plasmapheresis. It improved shortness of breath and proximal weakness although his bulbar symptoms persisted. He was discharged home on a tapering dose of steroids along with instructions to discontinue immunotherapy.

After discharge, the patient experienced two separate relapses with worsening diplopia, partial ophthalmoparesis, ptosis, dysphagia, dysphonia, and weakness requiring inpatient care. After his third relapse, he was started on rituximab which provided significant improvement in eyelid ptosis, dysphagia, and generalized weakness although he continued to suffer from binocular diplopia. He has been following with us for two years and has had no hospitalizations for any new neurological deficits. He is doing well without the requirement of any daily steroids.

## Discussion

Checkpoint inhibitors are monoclonal antibodies that have revolutionized the treatment of various solid and hematological malignancies [3]. They act against cytotoxic T lymphocyte antigen 4 (CTLA4), programmed cell death 1 (PD1) pathway, or programmed cell death ligand -1 (PDL-1). Ipilimumab, an anti-CTLA4 monoclonal antibody was the first FDA-approved immune checkpoint inhibitor in 2011 [4]. Nivolumab is an anti-PD-1 monoclonal antibody. As the use of checkpoint inhibitors is increasing so is the knowledge about their adverse effects on different organ systems of the body. The incidence of adverse events from checkpoint therapy is as low as 1% and as high as 90% [1,5]. There is evidence of pulmonary, cardiovascular, renal, endocrine, and neurological toxicities from their use. Thyroid dysfunction is the most common endocrinopathy observed, and was seen in our patient. [6].

Regarding neurological adverse effects, both central and peripheral neurotoxicity can be seen. The occurrence of neurological deficits is more likely to happen when combination therapy is used [2]. Neurological adverse effects are a rare but often severe complication of the use of checkpoint inhibitor treatment. They occur in about 1-5% of patients treated with checkpoint inhibitors [5]. Neurotoxicity seems to occur early with a median onset time of 6 weeks from the start of checkpoint inhibitor therapy [2]. Central neurotoxicity from these inhibitors can cause immune-mediated encephalitis, aseptic meningitis, and posterior reversible encephalopathy syndrome. Patients often present with altered mental status, headache, expressive or receptive aphasia, and motor or sensory changes [7,8].^ ^Peripheral nervous system (PNS) disorder side effects include neuropathy, myasthenia gravis, myasthenic syndrome, and myopathy. While affecting the peripheral nervous system, checkpoint inhibitors can cause mild sensorimotor peripheral neuropathies which are reversible after stopping the offending drug. They can also present as acute Guillain Barre Syndrome, myasthenia gravis, or myositis [7,9]. Patients usually present with progressive motor weakness, cranial neuropathies, ptosis, diplopia, or dysphagia.

Usually, the diagnosis of checkpoint immunotherapy neurotoxicity is made by ruling out other common etiologies which may present similarly and by assessing the timeline of the therapy being started in congruence with the onset of symptoms. Apart from routine toxic, metabolic, infectious, and neoplastic workup, EMG/NCS, lumbar puncture (LP)**, **and MR Brain are obtained to narrow the diagnoses. Stopping the offending drug, and intravenous pulse steroids are the mainstay of the treatment. If symptoms persist then intravenous immunoglobulins and plasmapheresis can be used. The majority of patients (70%) respond to the intravenous steroids [2,7].

There are no guidelines currently for managing patients who do not respond to steroids, IVIG, or PLEX. Such patients anecdotally are treated with the next line of immunosuppressive drugs like rituximab. There are few case reports demonstrating the benefits of rituximab in refractory myasthenia gravis from ICI [10]. A review of nine cases of ICI-related adverse effects treated with rituximab done by Deftereos et al. showed that rituximab was effective only in 67% of cases of adverse effects from ICI [11]. The response from rituximab may vary depending on if the given adverse effect pathology is B-cell dependent or not. 

## Conclusions

Our case demonstrates the importance of awareness of the toxicity of checkpoint inhibitors in patient care and the effectiveness of rituximab in refractory neurological adverse effects from checkpoint inhibitor therapy. Since it has only been reported a few times in the past, further trials are needed to prove its efficacy in such cases.
